# Intraoperative Speckle Variance Optical Coherence Tomography for Tissue Temperature Monitoring During Cutaneous Laser Therapy

**DOI:** 10.1109/JTEHM.2019.2943317

**Published:** 2019-09-25

**Authors:** Shoujing Guo, Shuwen Wei, Soohyun Lee, Mary Sheu, Sewon Kang, Jin U. Kang

**Affiliations:** 1 Electrical and Computer Engineering DepartmentJohns Hopkins University 1466 BaltimoreMD21218USA; 2 Department of DermatologyJohns Hopkins Medicine - Green Spring StationLuthervilleMD21093USA; 3 Department of DermatologyThe Johns Hopkins Hospital 1501 BaltimoreMD21287USA

**Keywords:** Cutaneous laser therapy, speckle variance OCT, tissue temperature monitoring, thermal modeling of tissue

## Abstract

*Background:* Tissue temperature monitoring during cutaneous laser therapy can lead to safer and more effective treatments. In this study, we investigate the use of speckle variance optical coherence tomography (svOCT) to monitor real-time temperature changes in the excised human skin tissue sample during laser irradiation. *Methods:* To accomplish this, we combined the pulse laser system with a reference-based svOCT system. To calibrate the svOCT, the ex-vivo human skin samples from three individuals with tissues collected from the arm, face, and back were heated with 1-degree increments. Additionally, linear regression was used to extract and evaluate the linear relationship between the temperature and normalized speckle variance value. Experiments were conducted on excised human skin sample to monitor the temperature change during laser therapy with a svOCT system. Thermal modeling of ex-vivo human skin was used to numerically simulate the laser-tissue interaction and estimate the thermal diffusion and peak temperature of the tissue during the laser treatment. *Results and Conclusion:* These results showed that normalized speckle variance had a linear relationship with the tissue temperature before the onset of tissue coagulation (52°) and we were able to measure the rapid increase of the tissue temperature during laser therapy. The result of the experiment is also in good agreement with the numerical simulation result that estimated the laser-induced peak temperature and thermal relaxation time.

## Graphical abstract

Tissue temperature monitoring during cutaneous laser therapy can lead to safer and more effective treatments. In this study, we investigate the use of speckle variance optical coherence tomography (svOCT) to monitor real-time temperature changes in the excised human skin tissue sample during laser irradiation. To accomplish this, we combined the pulse laser system with a reference-based svOCT system. To calibrate the svOCT, the ex-vivo human skin samples from three individuals with tissues collected from the arm, face, and back were heated with 1-degree increments. Additionally, linear regression was used to extract and evaluate the linear relationship between the temperature and normalized speckle variance value. Experiments were conducted on excised human skin sample to monitor the temperature change during laser therapy with a svOCT system. Thermal modeling of ex-vivo human skin was used to numerically simulate the laser-tissue interaction and estimate the thermal diffusion and peak temperature of the tissue during the laser treatment. These results showed that normalized speckle variance had a linear relationship with the tissue temperature before the onset of tissue coagulation ($52^{\circ}$) and we were able to measure the rapid increase of the tissue temperature during laser therapy. The result of the experiment is also in good agreement with the numerical simulation result that estimated the laser-induced peak temperature and thermal relaxation time.

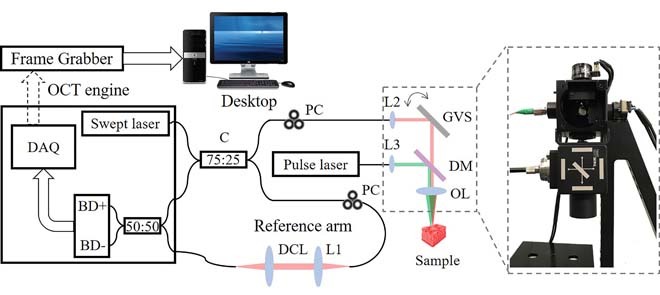

## I. Introduction

Cutaneous laser therapy has been widely used to treat skin conditions such as port wine stains, acne scars, photodamage, and wrinkles over the past 40 years [1]. The principle of laser therapy is based on selective photothermolysis, which allows selective laser heating of the target tissue with minimal or no damage to surrounding tissue structures [2]. In addition to select wavelengths, most laser therapy systems have other system parameters such as spot size, pulse duration, and frequency. To avoid either overheating that can cause collateral damage or underheating the tissue, which can lead to a lack of therapeutic effect, it is essential to use the appropriate parameters. Currently, for the most laser system, selection of treatment parameters is a manual process and is reliant on both the laser operator’s experience and knowledge of clinical endpoints [3]. Even for systems that have pre-programmed settings based on skin type and other patient criteria, the laser operator must have sufficient knowledge and judgment to accurately assess the patient’s skin type and its interaction with the laser. Given that laser operators have widely varying degrees of competence and training, more objective measures of laser-tissue interaction would allow for safer and more efficacious treatments.

In the past, methods for real-time tissue temperature monitoring have been proposed and investigated. A thermocouple or fiber sensor has shown to be an effective method that can monitor temperature accurately, but such an approach is invasive and only can be applied to a small area [4]. Magnetic resonance imaging (MRI) and Ultrasound (US) imaging-based methods provide a noninvasive way to monitor temperature change [5]–[7]. However, MRI is expensive and slow for real-time imaging, and the US has very limited accuracy due to the low-temperature sensitivity. Recently, Wu *et al.* [8] proposed photoacoustic imaging for precision tissue temperature monitoring. Nevertheless, the imaging speed is one frame per second, which is incapable of capturing the rapid temperature change caused by a pulsed laser. Thus, there is a need for a noninvasive monitoring method that can detect rapid temperature change over a wide treatment area.

Optical coherence tomography (OCT) [9], a noninvasive 3-D imaging modality with micrometer resolution has been used for dermatologic research as well as in clinical settings since 1997 [10], [11]. In dermatology, OCT systems have been used to study various skin diseases and treatment [12]–[15]. Themstrup *et al.* [16] used OCT to identify non-melanoma skin cancer. Tsai *et al.* [17] monitored the wound healing process of human skin by OCT. OCT systems have also been used for tissue temperature studies. For instance, Greco *et al.* [18] estimated the collapse temperature of pharmaceutical formulations under a freeze and drying process. Koinzer *et al.* [19] studied the temperature rise during laser irradiation of the retina. Seevaratnam *et al.* [20] used envelop statistics to quantify the relationship between OCT signals and temperature change of a phantom under heating.

OCT is based on coherent imaging and as a consequence, the images contain speckle patterns. Although speckle is often viewed as a noise source, it carries information. The change of speckle pattern in the OCT images of biological tissue is an indication of motion of the tissue molecules, and this can be detected and quantitatively analyzed by calculating speckle variance between frame or lines in OCT images [21]. Speckle variance OCT (svOCT) has been developed and used for the visualization of microvasculature [22], [23]. Using a similar idea, Lee *et al.* [24] were able to monitor protein denaturation and coagulation process with svOCT, which was based on the speckle pattern change due to tissue temperature change. Also, Lee *et al.* [25] used an in-house built svOCT system integrated into a commercial ophthalmic laser system and analyzed the relationship between the average laser energy and peak intensity of the svOCT image during selective retina therapy. But both papers didn’t reveale the direct relationship between speckle variance values and temperature and cannot perform high-speed temperature assessment.

In this study, we proposed to use svOCT to monitor the skin tissue temperature change during cutaneous pulsed laser therapy. This approach is based on previous work that shows the speckle variance values in OCT images directly relates to the tissue temperature change [24]. Thus, it is expected that the speckle variance value can represent the thermal induced molecular motion even when heated rapidly using a short-pulsed laser and still maintain a linear relationship with peak tissue temperature. To investigate this hypothesis, we built a test platform that combines a swept-source OCT system with a short-pulsed laser system and matches their focal plane to perform the in-vivo temperature monitoring during laser treatment. To correlate the tissue temperature with normalized speckle variance values obtained from B-scan OCT images, the samples from three individuals with tissues collected from the arm, face, and back were heated in a water bath by a hot plate in 1-degree increments, and normalized speckle variance images were calculated at different temperature levels. Then, we performed the linear regression to quantify the relationship between normalized speckle variance value and tissue temperature. We computed cross-correlation between the measured and expected speckle variance values to evaluate the results. The repeatability test was performed during the calibration process by choosing several measurements close approximately to each other on the sample tissue from the back. Once we obtained the relationship between the tissue temperature and the speckle variance values, we performed the experiment using a pulsed laser. The excised human skin sample was irradiated with a microsecond pulse laser and tissue temperature change during therapy was deduced from the speckle imaging. To verify the result, the spatial and temporal temperature distribution of the skin tissue was numerically modeled and evaluated.

## II. Methods and Procedures

### A. SVOCT System Setup

**FIGURE 1.**
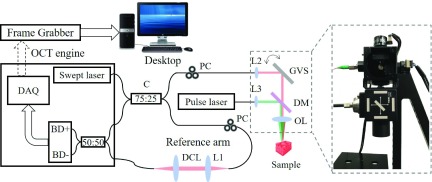
System configuration: BD+, BD−, balance detector; DAQ, data acquisition board; C, 75:25 broadband coupler; L1, L2, L3, achromatic collimators; DCL, dispersion compensation lens; PC, polarization controller; GVS, galvanometer pairs (for simplicity only show one mirror); DM, dichroic mirror; OL, objective lens.

The system setup is shown in Fig.1. To perform intraoperative speckle variance imaging, an in-house developed swept source optical coherence tomography system was used. The system uses a commercial swept source OCT Engine (Axsun Technologies, Inc.) with ~1060 nm center wavelength, full sweeping bandwidth of ~100 nm and 100 kHz sweep rate. The laser output was split into sample arm and reference arm by a 75:25 optical fiber coupler (TW1064R3A2A, Thorlabs) and polarization controllers (FPC020, Thorlabs) were used on both arms to match the polarization state. The output laser from the sample arm was focused on a tissue sample with an objective lens (AC127-019-C, Thorlabs). The reference and signal then were combined by a 50:50 coupler and detected by a balanced detector embedded in the OEM engine. The resultant interferometric signal is processed by a camera-link data acquisition board with a built-in high-speed digitizer (500 MSPS, 12-bit resolution). The gavlo scanner (GVS001, Thorlabs) was used for B-scan OCT imaging and synchronize with the laser trigger signal by a multifunction I/O device (PCI-E 6221, National Instruments).

The sampled spectrum OCT data was captured by a frame grabber (PCI-E 1433, National Instruments) and processed in parallel with a discrete graphics card (Quadro K4200, NIVIDA) for high-speed real-time imaging processing, including fast Fourier transform (FFT), background noise subtraction and speckle variance calculation. The system was set to process 1024 pixels per A-scan and 1024 A-scans per B-scan. The center wavelength of 1060 nm was chosen to achieve higher resolution with a reasonable imaging depth inside the tissue. The measured lateral scanning resolution was $12~\mu \text{m}$ and the axial resolution was $4.5~\mu \text{m}$ in the tissue, with a measured imaging depth of 850 microns. The 6 dB roll-off of our current system was measured to be 6 mm in the air. The output power from the sample arm was ~2.5 mW. A customized user interface was designed and programmed through C++/C# (Visual Studio 2010, Microsoft).

To simulate the tissue during cutaneous laser therapy and to heat the tissue, we used a frequency-doubled Nd:YLF pulse laser system (R:GEN, Lutronics) as the therapeutic laser. The system can provide up to 200 micro joules of energy per pulse for the treatment with the center wavelength of 532 nm, 100 Hz repetition rate and $1.7~\mu \text{s}$ pulse duration. A dichroic mirror (DMLP735B, Thorlabs) was used to combine the pulse laser beam and the OCT swept laser beam. The beams were aligned such that the pulse laser irradiation area was at the center of each B-scan image, which makes it easy to visualize the laser therapy on the tissue sample.

### B. Temperature Calibration Setup

**FIGURE 2.**
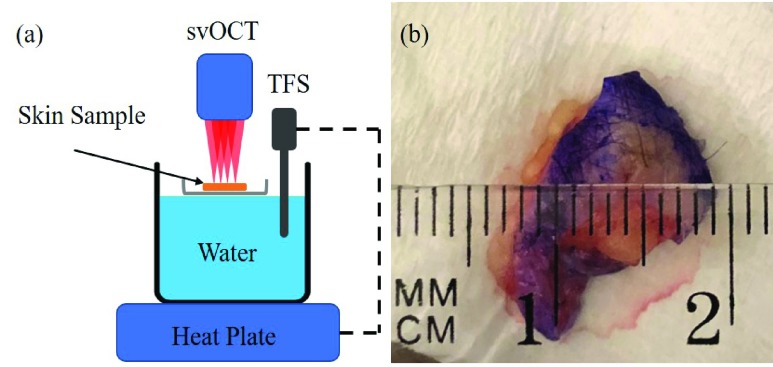
(a) System setup for heating the skin tissue by 1-degree increment, TFS, temperature feedback sensor. (b) “Dog ear” skin sample from the back with 1mm diameter.

To quantify the relationship between the normalized speckle variance value and tissue temperature, an excised human skin sample was put on a heating pot and heated in a water bath with a hot plate (MS-H280-Pro, Scilogex) as shown in Fig. 2(a). The temperature is monitored and controlled by a temperature feedback sensor (PT1000, Scilogex) with an accuracy of ± 0.5 °. The skin sample was heated from 25°C to 67°C with 1°C increment. Standard OCT B-scan images were saved and processed to deduce normalized speckle variance values, and the result was analyzed to obtain its relationship with the corresponding temperature.

The excised human skin sample (“dog ear”) shown in Fig. 2(b) was obtained from an 86-year-old female patient’s back who underwent excision with margins of a non-melanoma skin cancer of the lower extremity. The dog ears are triangles of redundant skin that are often excised during excisional skin surgery to achieve a smooth contour without topographical elevations or puckering. They are typically composed of normal (non-cancerous) skin. The tissue is a full-thickness skin sample that includes epidermis, dermis, and portion of subcutaneous fat. The dog ear was stored at ambient temperature on normal saline-soaked gauze for a short time before it was used.

To further investigate the variability among different human tissues we performed measurements using ex-vivo skin samples from three individuals with tissues collected from the arm, face and back with similar sample size. As for verifying the repeatability of the svOCT method, we performed several measurements close proximity to each other in space with the same region of interest’s (ROI) pixels. This was done since repeating the measurements on a same sample changes the tissue characteristics.

### C. Normalized Speckle Variance Calculation and Linear Regression

To obtain the normalized speckle variance values, we calculated the interframe normalized speckle variance ($SV_{ijk}$) of the certain number ($N$) of B-scan image using the following equation:

\begin{align*} SV_{ijk}=&\frac {1}{N}\sum \limits _{i=1}^{N} {\left [{ {\frac {I_{ijk} -\frac {1}{N}\sum \nolimits _{i=1}^{N} {I_{ijk}} }{\frac {1}{N}\sum \nolimits _{i=1}^{N} {I_{ijk}}}} }\right]}^{2} \\=&\frac {1}{N}\sum \limits _{i=1}^{N} {\left [{ {\frac {I_{ijk} -I{}_{mean}}{I_{mean}}} }\right]}^{2}\tag{1}\end{align*}

$N$ is the number of B-scan images used in normalized speckle variance calculation. $I_{ijk}$ represents the OCT signal intensity at pixel (*j,k*) in the $i$’th B-scan image. $j$ and $k$ represent the lateral and depth position in the B-scan image. $I_{mean} $ is the averaged pixel intensity of the total $N$ B-scan images at the same point. The normalized method divides the variance by its mean values to reduce typical noises in the OCT systems such as shot noise, excess intensity noise, thermal noise, and flicker noise and laser power fluctuations effects on the linear regression. In this paper, the averaged B-scan images number $N$ used was 50. This is due to the fact that the longest period in which the tissue temperature is relatively stable was measured to be 0.5 seconds and the large data set results in a better calibration. The selected ROI were $700\times50$ pixels along the X- and Z-directions for the temperature calibration. And the selected ROIs for repeatability test were $200\times50$ pixels in X- and Z-direction.

The linear regression was performed to quantify the relationship between the normalized speckle variance value and the tissue temperature before the tissue coagulation due to excessive heating. To determine the linear regression results, the linear cross-correlation coefficient ($r$) of the estimated average normalized speckle variance values of ROI (*SV*) and the measured value ($S{V}'$) was calculated using the following equation:

\begin{equation*} r=\frac {\sum \nolimits _{q=1}^{M} {\left ({{SV_{q} -\overline {SV}} }\right)\left ({{SV_{q}^{\prime }-\overline {S{V}'}} }\right)}}{\sqrt {\sum \nolimits _{q=1}^{M} {\left ({{SV_{q} -\overline {SV}} }\right)^{2}}} \sqrt {\sum \nolimits _{q=1}^{M} {\left ({{SV_{q}^{\prime }-\overline {S{V}'}} }\right)^{2}}}}\tag{2}\end{equation*}

Here, $\overline {SV} $ and $\overline {S{V}'} $ are the average values of the measured normalized speckle variance value and the estimated value. $M$ is the number of points in the dataset and $q$ is the indices of normalized speckle variance data.

### D. Laser Therapy Experimental Conditions

The human skin sample from the back with similar size was used for the laser therapy experiment. The sample was placed in a beaker under the svOCT/pulse laser system. The energy of the pulse laser system was set to approximately 100 micro joules of energy. To monitor the tissue temperature change during the whole therapy, the svOCT started recording B-scan images and normalized speckle variance images before the pulse laser was on. After 10 seconds, the pulse laser was turned on for 0.1 seconds during which ten laser pulses irradiated the center of the tissue. The svOCT system kept running for another 10 seconds while the tissue cooled down. The images were analyzed by using the calibrated coefficients between speckle variance value and temperature to map the temperature change during the laser therapy. Two ROIs were chosen, one was on laser irradiated area with a size of $30\times100$ pixels along the X- and Z-directions and the other was the adjacent area outside irradiation with the same size.

### E. COMSOL Simulation Setup

COMSOL Multiphysics software (COMSOL 5.4) was used to conduct spatial and temporal temperature modeling of the skin tissue sample during the cutaneous laser therapy to verify the result we acquired from the svOCT system. Conduction heat flow is governed by classical Pennes bioheat transfer equation [26]:

\begin{equation*} \rho C\frac {\partial T}{\partial t}=K\nabla ^{^{2}}T+Q_{r} +\textrm {W}_{b} C_{b} (T_{b} -T)\tag{3}\end{equation*}

where $\rho $ is tissue density, $C$ is tissue-specific heat and $K$ is the thermal conductivity of the tissue. The term $W_{b}C_{b}(T_{b}{\text{-}}T)$ represented the blood perfusion. Here, $W_{b }$ is the blood perfusion rate, $C_{b}$ is the specific heat of blood, $T_{b}$ is the temperature of artery and $T$ is the tissue temperature. According to the equation above, the blood perfusion will cause the temperature change slower during the laser irradiation since the blood will take away heat. Since the ex-vivo human skin samples didn’t have blood perfusion, this term can be ignored by setting $W_{b}$ to zero. The laser source term $Q_{r}$ of Gaussian laser-beam profile can be described as (4) using the cylindrical coordinate [27].

\begin{align*} Q_{r}(r,z,t)=&-dI/dz \\=&\left [{\mu _{t}+0.5\mu _{s}(r/\omega (z)^{2})}\right]I(r,z,t)\tag{4}\\ I(r,z,t)=&I_{0}~\text {exp}(-2r^{2}/\omega ^{2}_{0})\ast f(t)\tag{5}\end{align*}

Here, $I_{0}$ is the fluence rate and $\omega _{0}$ is the radius of the laser beam where the intensity decays to 1/$e^{2}$. $\mu _{t} = \mu _{a} +\mu _{s}, \mu _{s}$ is the tissue scattering coefficient and $\mu _{a}$ is the tissue absorption coefficient at a certain wavelength. $f(t)$ here is the time dependence function used to simulate the pulse laser irradiation.

Due to the symmetry of the experiment setup, the simulation was simplified and calculated in a 2D symmetry cylindrical coordinate. The dimension of the tissue sample in the simulation was set to 25 (width)$\times 10$ (height) mm. Since the thermal properties of the skin tissue is a function of temperature, highly accurate thermal modeling is difficult to achieve [28]. Thus, we assumed the parameters are consistent during our simulation. The parameters of tissue thermal properties are calculated from the following equations as a function of water content ($W$) [29], [30]:

\begin{align*}\rho=&(6.16\times 10^{-2}w+0.938)^{-1}\tag{6}\\ C=&2.5W+1.7\tag{7}\\ K=&\rho \times 10^{-2}(0.454W+0.174)\tag{8}\end{align*}

According to the previous study, the water content in the dermis is around 70% [28], and ex-vivo skin sample is considered to have lower water content [31]. Here, the water content in our ex-vivo skin samples were slightly dryer than in in-vivo skin and based on the reduced density of the sample we estimated it to be approximately 55%. The scattering and absorption coefficient were chosen based on the previous study at the wavelength of 527nm [32]. Table 1 summarize all the parameters for the simulation.

**TABLE 1:** Parameters for Laser and Modeling of Skin Tissue

${W}$-water content	55%
$\rho $-tissue density	1.03 g/cm^3^
$C$-tissue specific heat	3.075 J/(g$\cdot $K)
$K$-thermal conductivity of tissue	$4.4\times 10^{\mathrm {-3}}$ W/(cm$\cdot $K)
$T_{0}$-initial temperature	301.15 K
$I_{0}$-fluence rate	$4.53\times 10^{\mathrm {5 {}}}\text{W}$/cm^2^
$\mu _{a}$-absorption coefficient at 527 nm	2.0 cm^−1^
$\mu _{s}$-scattering coefficient at 527 nm	100 cm^−1^
$\omega _{0}$-radius of laser beam	$75~\mu \text{m}$

## III. Results

### A. Normalized Speckle Variance Values as a Function of Tissue Temperature

**FIGURE 3.**
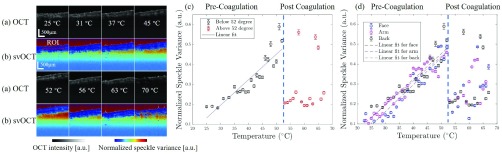
(a) Normal intensity-based OCT images and (b) Corresponding svOCT images of “dog-ear” sample from the back during 8 different heating temperatures, (c) Linear regression between normalize speckle variance and tissue temperature before coagulation, (d) Calibration results on “dog-ear” sample from three different sites.

Fig. 3 shows (a) the standard intensity-based OCT images and (b) corresponding svOCT images of the dog-ear sample from the back during OCT temperature calibration. Here, we show the images at eight different temperatures, 25°C, 31°C, 37°C, 45°C, 52°C, 56°C, 63°C, and 70°C respectively. As the temperature increased, the standard B-scan images in Fig. 3(a) didn’t change much when compared to other images in Fig. 3(a). However, the svOCT images when compared to other images in Fig. 3(b), one can see that the normalized speckle variance values in the ROI increased until it reached 52°C and its values decreased significantly after 52°C, which was the threshold temperature for the tissue coagulation. Applying the linear regression to the normalized speckle variance values before 52°C, we obtained the following relationship as a function of tissue temperature.

\begin{equation*} S{V}'_{back} =0.0115T-0.1877,T\in \left ({{25^{\circ }C,52^{\circ }C} }\right)\tag{9}\end{equation*}

$S{V}'_{back} $ here is the estimated average normalized speckle variance values of ROI and $T$ is the measured temperature. The calculated cross-correlation value was $r_{back}=0.9007$, which indicated the linear regression model fits well between the normalized speckle variance value and temperature before the onset of tissue coagulation. After 52°C, the tissue coagulated and caused the normalized speckle variance values decreased to 0.60 +/− 0.01 at 53°. These results are well matched to previously reported denaturation and coagulation of egg white protein monitored by svOCT [24].

Two more calibration results of dog-ear samples from the arm and the face were plotted in Fig. 3(d). It shows the normalized speckle variance values as a function of tissue temperature in certain ROIs. From both three results, we can clearly identify the two stages, pre-coagulation and post coagulation. It shows highly consistent results where the three calibration results share very similar trends before coagulation and the coagulation in both three cases occurred at 52 °C, followed by a huge decrease in normalized speckle variance value. The measured decreases were 54.3%, 68.7% and 65.5% in the dog-ear samples from the face, arm, and back. The calculated linear regression results were shown as below, and the calculated cross-correlation values were $r_{face}=0.9484$ and $r_{arm}=0.9521$. Therefore, it appears that unless the skin density and pigmentation are significantly different, there is no need for new calibration.

\begin{align*} S{V}'_{arm}=&0.0123T-0.1750,T\in \left ({{25^{\circ }C,52^{\circ }C} }\right) \tag{10}\\ S{V}'_{face}=&0.0136T-0.2473,T\in \left ({{25^{\circ }C,52^{\circ }C} }\right)\tag{11}\end{align*}

**FIGURE 4.**
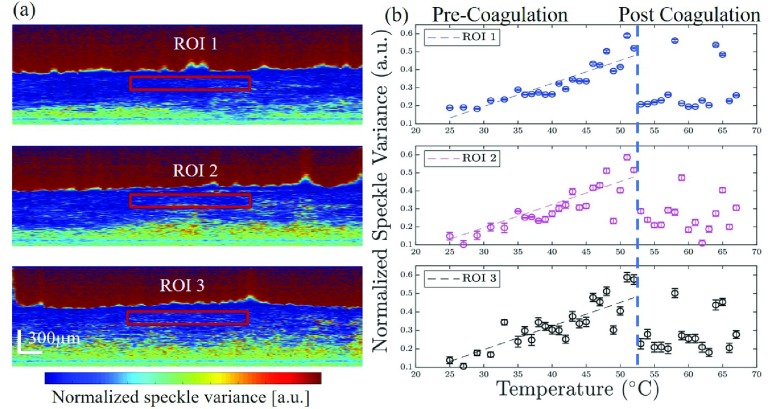
(a) Three ROIs on dog-ear sample from the back during calibration process, (b) Calculated normalized speckle variance values on three different ROIs.

As for the repeatability test, the speckle variance image from the calibration of dog-ear sample from the back was divided into three adjacent parts and three ROIs with same size were chosen from it as in Fig. 4(a) from top to bottom. The normalized speckle variance values in each ROI were plotted accordingly in Fig. 4(b) and in three ROIs, the normalized speckle variance values decreased 60.0%, 44.4%, 60.4% in ROI 1, 2 and 3 after 52 °. The calculated linear results for three ROIs were shown as below.

\begin{align*} S{V}'_{ROI1}=&0.0128T-0.1877,T\in \left ({{25^{\circ }C,52^{\circ }C} }\right) \tag{12}\\ S{V}'_{ROI2}=&0.0138T-0.2488,T\in \left ({{25^{\circ }C,52^{\circ }C} }\right) \tag{13}\\ S{V}'_{ROI3}=&0.0142T-0.2433,T\in \left ({{25^{\circ }C,52^{\circ }C} }\right)\tag{14}\end{align*}

To further investigate the repeatability and reliability of our proposed method, the intraclass correlation coefficient ($r_{icc}$) before coagulation was calculated as 0.9179, which indicated that normalized speckle variance values in three ROIs at the same temperature are consistent with each other well. The results matched well with each other and showed the repeatability of the svOCT measurement.

Based on the average fitting error of calibration results, the sensitivity of the proposed method was deduced and found to be approximately 3.2 °. However, this can be improved with a longer integration time and integrating over a larger pixel size that degrades the temporal and spatial resolution.

### B. Temperature Monitoring During Cutaneous Laser Therapy

**FIGURE 5.**
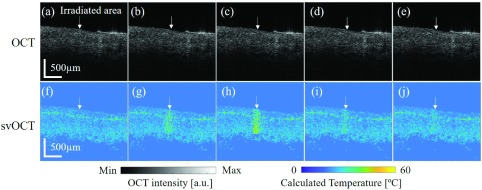
Normal intensity-based OCT images and corresponding svOCT images during cutaneous laser irradiation. (a) and (f) OCT images and normalized speckle variance images before the laser irradiation, (b), (c), (g) and (h) pulse laser irradiated on skin sample from the back, (d), (e), (i) and (j) pulse laser turned off.

Fig. 5. shows the intensity-based OCT images and corresponding normalized speckle variance OCT images during the high-power pulsed laser therapy. The white arrow indicates the irradiated area on the dog-ear sample from the back. The Fig. 5(a) and (f) show the OCT and speckle variance images before the pulse laser turned on and the speckle variance value was low as expected at this time. When the laser was turned on as shown in Fig. 5(b) and (g), the normalized speckle variance value of the irradiated area increased immediately, which indicated the temperature in that area increased rapidly. However, during this same time, one can see that the intensity-based OCT image remained the same. As multiple pulses laser irradiates the same spot, the speckle variance value kept increasing and the increased in the value can be seen inside the tissue as deep as $\sim 600~\mu \text{m}$ from the top of the sample. The other areas remained the same as before the laser irradiating. When the pulse laser was off as shown in Fig. 5(d), (e), (i) and (f), the speckle variance value started decreasing and came back to the normal value as the tissue cooled down to the room temperature.

**FIGURE 6.**
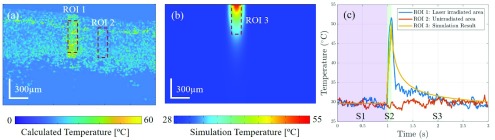
Analysis of ROIs’ temperature during cutaneous laser therapy. (a) Two selected ROIs on the svOCT image when pulse laser irradiated on skin sample from the back, ROI 1, laser irradiated area, ROI 2 unirradiated area in the neighbor, (b) 2D Simulation result by COMSOL when pulse laser irradiated, (c) Temperature change in three ROIs by time, S1, stage 1-before laser therapy; S2, stage 2-pulse laser irradiated on sample; S3, stage 3-pulse laser off.

Applying the linear regression result (9) to the normalized speckle variance images during the pulse laser irradiation, the temperature change was deduced from the speckle variance images. We chose two ROIs as shown in Fig. 6(a), one was on the laser irradiated area having a size of $30\times100$ pixels along the X- and Z-directions and the other was the adjacent area outside the irradiation with the same size. The result was plotted in Fig. 6(c), the tissue temperature in ROI 1 was calculated as 29.7 +/− 0.7 °C and 29.5 +/− 0.6 °C in ROI 2 at S1 before the pulse laser was turned on. S2, is the stage when the pulse laser was turned on and following the pulse shape the calculated temperature raised up to 51.7 °C in 0.1 seconds during which 10 pulses 10 milliseconds apart irradiated the tissue in ROI 1. Although, the skin temperature during the laser treatment depends on the type of procedure, skin condition, and laser setting. The similar temperature distribution was reported during the treatment for oculodermal melanosis and the highest temperature in their procedure reached around 52 °C [33]. At S3, the laser was turned off and the calculated temperature decreased quickly from 51.7 °C to 34.2 °C in 0.1 seconds in ROI 1. It took 1.5 seconds for the tissue to cool down from 34.2°C to 29.3°. During the whole laser irradiation process, the calculated temperature of ROI 2 remained at 29.7 +/− 0.7 °. The speckle variance values in ROI 1 during the therapy didn’t show any sudden drop pattern like in our temperature calibration process, which indicated that there was no coagulation inside the tissue.

### C. Simulation Results During Cutaneous Laser Therapy

Fig. 6(b) shows the result of 2D numerical simulation of the skin tissue model at the highest temperature. A similar size of ROI was chosen to compare the simulation and experimental result. The simulation result is plotted in Fig. 5(c) in yellow. Once the laser was turned on, the temperature in ROI 3 raised from 29 °C to 50.1 °C in 0.1 seconds. The peak temperature was similar compared to 51.7 °C from the svOCT. It took the tissue almost 2 seconds to cool down in the simulation. To further compare the result, the cross-correlation coefficient ($r$) between the two curves was calculated and we obtained $r =0.88$. Thus, the simulation result and temperature measured by svOCT shared the same trend and matches well with each other. However, we noticed the experimental temperature recovery was faster than in the simulation. In the simulation, the model used a uniform and smooth surface but, in reality, the tissue surface was not uniform and had a larger surface area. Another reason is that the water in tissue evaporates promoting a faster cooling process.

## IV. Conclusion

Our results showed that svOCT can be used to accurately and safely analyze the change in tissue temperature during the pulse laser irradiation. The calibration result showed that the normalized speckle variance had a linear relationship with the tissue temperature until the onset of tissue coagulation (52°). After the coagulation, the normalized speckle variance value becomes small and decrease rapidly. These results match well with the previous study on egg white protein denaturation and coagulation temperature monitoring by svOCT [24]. Also, our numerical simulation results are in good agreement with our experimental result. The normalized svOCT method can provide the laser therapy process with a high depth-resolution of 4.5 microns and high time-resolution of 10 milliseconds to capture the temperature change inside the tissue. The svOCT temperature monitoring is noninvasive and easy to integrate with a laser therapy system. Conversely traditional methods such as thermocouple are invasive and photoacoustic imaging method is relatively slow, with a time resolution in the order of 1 second. The proposed method was able to measure the rapid increase of the excised human skin temperature up to approximately 52°C when irradiated by the pulse laser. Above 52°C degrees, the tissue coagulated, and the normalized speckle variance decreased quickly. This is also one drawback of the system: it is hard to monitor the temperature once the tissue coagulates and this makes it impossible to do the calibration at the same spot on the patient. Future work will be focused on enlarging the test data sets of different human skin samples and performing the calibration and temperature monitoring during the clinical therapy process. The svOCT temperature monitoring methods could lead to safer and more effective laser treatments in dermatology and as a research tool, svOCT could further elucidate the effects of laser on skin structures in real-time in the future.
